# Recovery of 26 metagenome-assembled genomes from the phycosphere of the marine diatom *Skeletonema tropicum*

**DOI:** 10.1128/mra.01013-24

**Published:** 2025-03-25

**Authors:** Tian Xia, Jiayi Yang, Songze Chen, Huiquan Li, Shuaishuai Xu, Kangli Guo, Shengwei Hou

**Affiliations:** 1 Department of Ocean Science and Engineering, Southern University of Science and Technology 547494 , Shenzhen, Guangdong, China; 2 Shenzhen Ecological and Environmental Monitoring Center of Guangdong Province, Shenzhen, Guangdong, China; Montana State University, Bozeman, Montana, USA

**Keywords:** metagenome-assembled genomes, phycosphere, *Skeletonema*, marine diatom

## Abstract

Metagenome-assembled genomes (MAGs) were recovered from the phycosphere of marine diatom *Skeletonema tropicum*, which has been long-term maintained in artificial seawater. Most MAGs were found to be highly complete (>90%) with minimum contaminations (<5%), which could serve as reference genomes to investigate the interactions between *Skeletonema* and their phycosphere microbiota.

## ANNOUNCEMENT

Diatom-bacteria interactions have developed over 200 million years (1), forming complex relationships that facilitate nutrient exchange and biogeochemical cycling. These interactions are predominantly mutualistic, where bacteria obtain organic matter from diatoms, while diatoms benefit from essential nutrients like vitamins and remineralized nitrogen supplied by bacteria (1–3). Understanding the metabolic capacity of phycosphere microbiota and their interactions with host phytoplankton is vital for understanding the growth dynamics of marine phytoplankton and their contribution to global biogeochemical cycles.

Here, we report the recovery of 24 high-quality and two medium-quality metagenome-assembled genomes (MAGs) (4) from the hybrid metagenomic sequencing of a laboratory-maintained long-term culture of the marine diatom *Skeletonema tropicum*. This diatom was initially collected from the Pearl River Estuary in 2013 and has been maintained in non-axenic artificial seawater supplemented with f/2 medium at 20°C under 12:12 hour light/dark cycles. DNA was extracted and sequenced by Illumina and QitanTech Nanopore to obtain shotgun and long reads. The detailed information on DNA extraction, library preparation, and sequencing can be found in Guo et al. (5).

**Fig 1:**
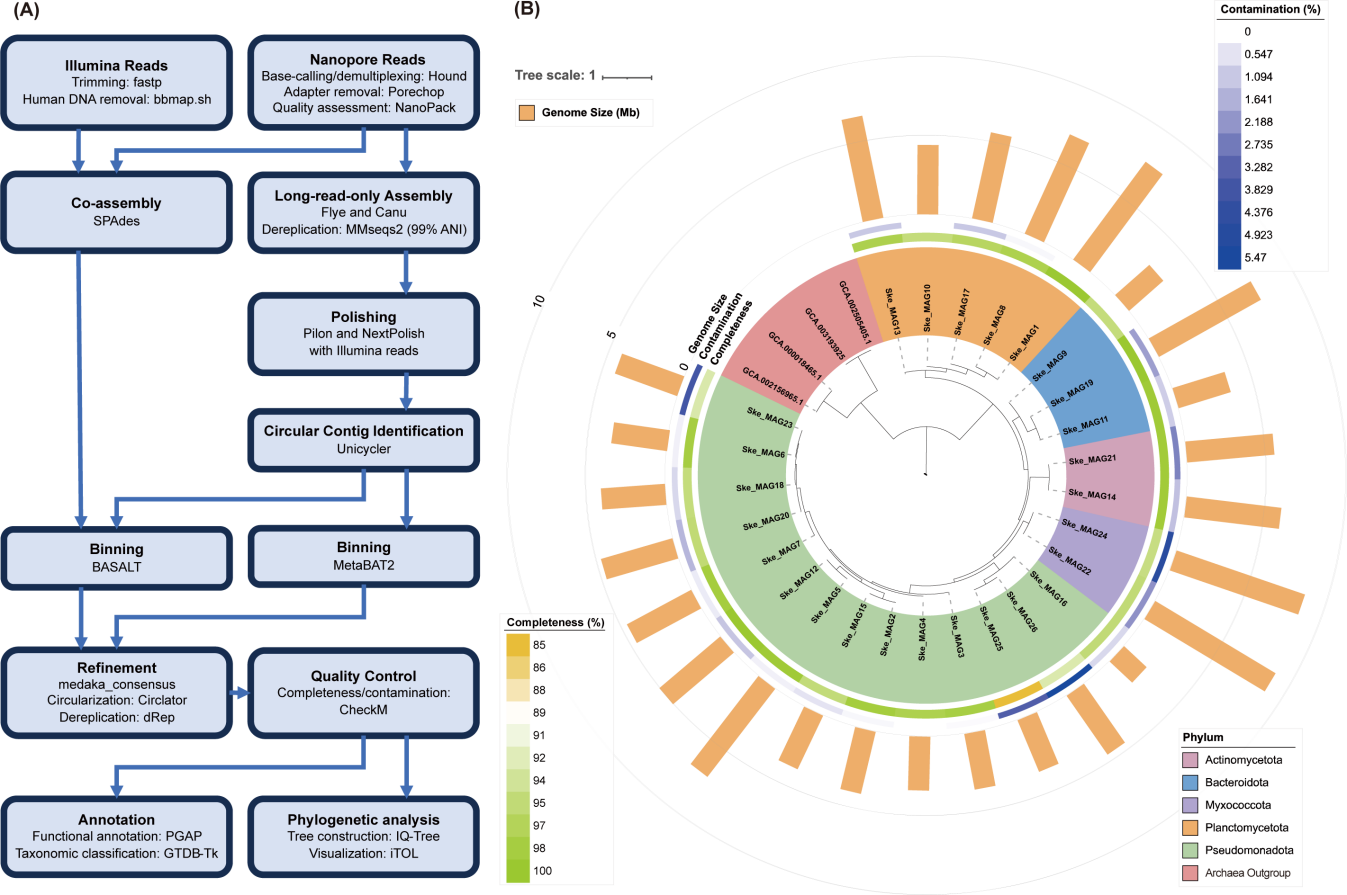
The computational workflow (**A**) and maximum likelihood phylogenetic tree (**B**) of the 26 MAGs recovered from the phycosphere of *S. tropicum*. Software versions can be found in the main text. Four archaea genomes (two MGI and two MGII) were used as the outgroup to root this phylogenomic tree. Shading colors represent the affiliated phyla of these MAGs. Detailed MAG genomic size, completeness, contamination, N50, and contig number information can be found in Table 1.

As shown in Fig. 1A, Illumina reads were first trimmed using fastp v0.19.5 (6). Human genome contaminations were removed using bbmap.sh (minid = 0.95, maxindel = 3, bwr = 0.16, bw = 12, quickmatch, fast) (http://sourceforge.net/projects/bbmap). Nanopore reads were base-called and demultiplexed using Hound v1.1 (Qitan Technology Co.). Adapters were removed using Porechop v0.2.4 (7), and the sequence quality was assessed using NanoPack v1.25.0 (8). Nanopore and Illumina reads were co-assembled by SPAdes v3.15.5 (-k 21,33,55,77,99,127) (9). Besides, the Nanopore reads were assembled by Flye v2.9 (10) and Canu v2.2 (11) and dereplicated by MMseqs2 (12) at 99% average nucleotide identity (ANI). The dereplicated contigs were then polished using Pilon v1.24 (13) and NextPolish v1.4.0 (14) with Illumina reads, and Unicycler v0.4.8 (15) was used to identify and clean circular contigs from the polished assemblies. MetaBAT2 v2.12.1 (16) was used for metagenomic binning based on coverage derived from Nanopore read mapping using minimap2 v2.22 (17). BASALT (18) was also used for binning based on the coverage of Illumina and Nanopore reads, and medaka_consensus (https://github.com/nanoporetech/medaka) was used to make consensus MAGs. MAGs were further circularized by Circlator (19) and dereplicated into unique MAGs using dRep v3.4.0 (-comp 50 -con 10) (20) at 98% ANI.

In total, 132 million clean short reads (39.74 Gbp) and 1.76 million clean long reads (4.6 Gbp raw data) were generated by Illumina and Nanopore sequencing, respectively (5). Twenty-six MAGs were recovered from the hybrid sequencing and metagenomic binning (Table 1). The completeness and contamination of these MAGs were estimated using CheckM v.1.2.1 (21), and taxonomy was classified using GTDB-Tk v2.1.1 (22) with the database GTDB v214. The 26 MAGs are affiliated with five bacterial phyla. A maximum likelihood phylogenetic tree was constructed using IQ-Tree v1.6.12 (-m LG+R10 -B 1000 -bnni -T AUTO -mem 500G) (23) and visualized using the iTOL Web server (https://itol.embl.de, Fig. 1B). Genomic annotation was done using PGAP (24). All tools described above were run with default parameters unless otherwise noted. The availability of these MAGs may facilitate studies of diatom-bacterial interactions in the ocean.

**TABLE 1:** Assembly and genomic information of the 26 MAGs

MAG name	Length (Mb)	Completeness (%)	Contamination (%)	N50 (Mb)	Contig numbers	NCBI GenBank accessions
Ske_MAG1	7.70	99.93	0	0.57	28	JBKAHA000000000
Ske_MAG2	4.03	98.8	0.2	1.00	16	JBKAGZ000000000
Ske_MAG3	3.58	98.32	0.16	3.58	1	JBKAGY000000000
Ske_MAG4	3.41	97.61	0	0.57	13	JBKAGX000000000
Ske_MAG5	7.03	99.31	0.32	0.31	37	JBKAGW000000000
Ske_MAG6^*a*^	3.57	98.51	0.32	3.21	3	CP096147.1
Ske_MAG7	4.61	98.7	0.4	0.28	19	JBKAGV000000000
Ske_MAG8	6.89	97.63	0.19	0.24	48	JBKAGU000000000
Ske_MAG9	2.88	95.46	0	1.67	6	JBKAGT000000000
Ske_MAG10	4.39	95.45	0	0.12	52	JBKAGS000000000
Ske_MAG11	3.45	99.34	0.99	1.02	6	JBKAGR000000000
Ske_MAG12	5.04	100	1.19	1.37	16	JBKAGQ000000000
Ske_MAG13	6.59	97.73	1.14	6.59	1	JBKAGP000000000
Ske_MAG14	6.03	99.15	1.28	0.14	76	JBKAGO000000000
Ske_MAG15	3.75	95.59	0.6	0.05	135	JBKAGN000000000
Ske_MAG16	1.95	95.64	0.78	0.04	95	JBKAGM000000000
Ske_MAG17	5.57	96.67	1.11	0.42	22	JBKAGL000000000
Ske_MAG18	4.06	95.41	0.81	0.13	56	JBKAGK000000000
Ske_MAG19	7.39	100	2.02	0.18	88	JBKAGJ000000000
Ske_MAG20	4.70	95.76	1.52	0.23	37	JBKAGI000000000
Ske_MAG21	5.54	99.15	2.56	0.87	15	JBKAGH000000000
Ske_MAG22	8.87	95.65	2.28	0.36	53	JBKAGG000000000
Ske_MAG23	4.18	93.36	3.78	0.06	199	JBKAGF000000000
Ske_MAG24	8.58	95.16	4.7	0.17	83	JBKAGE000000000
Ske_MAG25^*b*^	3.54	85.47	3.35	0.01	693	JBKAGD000000000
Ske_MAG26^*b*^	4.53	93.28	5.47	0.01	525	JBKAGC000000000

^*a*^ Ske_MAG6 was previously reported by Guo et al. (5), which was recovered from the same phycosphere sample, following the same methods of culturing, DNA isolation, library preparation, sequencing, and data analysis.

^*b*^ Two medium-quality MAGs.

## Data Availability

The SRA accession numbers of the Illumina and Nanopore reads are SRR30551005 and SRR30565972, respectively. MAGs and genomic annotations have been deposited at the NCBI GenBank database under the umbrella BioProject accession number PRJNA827970.
